# Comparison and Determination of the Content of Mosapride Citrate by Different qNMR Methods

**DOI:** 10.3390/ijms251910442

**Published:** 2024-09-27

**Authors:** Xiaofang Lian, Yiran Li, Limin Zuo, Xuejia Zhao, Huiyi Liu, Yongsheng Gu, Qingying Jia, Jing Yao, Guangzhi Shan

**Affiliations:** 1 Institute of Medicinal Biotechnology, Chinese Academy of Medical Sciences & Peking Union Medical College, Beijing 100050, China; xiaofanglian0365@163.com (X.L.); liyiran@imb.pumc.edu.cn (Y.L.); zuo0607@163.com (L.Z.); zhaoxuejiajia@163.com (X.Z.); huiyiliu1227@163.com (H.L.); gys0525@163.com (Y.G.); qyjia1130@163.com (Q.J.); 2 College of Pharmacy, Xinjiang Medical University, Urumqi 830017, China; 3 Institute for the Control of Chemical Drugs, National Institutes for Food and Drug Control, Beijing 100001, China

**Keywords:** mosapride citrate, quantitative NMR, ^1^H NMR, ^19^F NMR, method optimization, method development, method validation, comparison of methods

## Abstract

As a salt-type compound, mosapride citrate’s metabolism and side effects are correlated with its salt-forming ratio. Several techniques were developed in this work to compare various quantitative nuclear magnetic resonance (qNMR) methodologies and to quantitatively examine the content of raw materials. Among the qNMR techniques, methods for ^1^H NMR and ^19^F NMR were developed. Appropriate solvents were chosen, and temperature, number of scans, acquisition time, and relaxation delay parameter settings were optimized. Maleic acid was chosen as the internal standard in ^1^H NMR, and the respective characteristic signals of mosapride and citrate were selected as quantitative peaks. The internal standard in ^19^F NMR analysis was 4,4′-difluoro diphenylmethanone, and the distinctive signal peak at −116.15 ppm was utilized to quantify mosapride citrate. The precision, repeatability, linearity, stability, accuracy, and robustness of the qNMR methods were all validated according to the ICH guidelines. By contrasting the outcomes with those from high-performance liquid chromatography (HPLC), the accuracy of qNMR was assessed. As a result, we created a quicker and easier qNMR approach to measure the amount of mosapride citrate and evaluated several qNMR techniques to establish a foundation for choosing quantitative peaks for the qNMR method. Concurrently, it is anticipated that various selections of distinct quantitative objects will yield the mosapride citrate salt-forming ratio.

## 1. Introduction

Mosapride citrate tablets are a novel gastrointestinal motility drug [1,2,3] which promote gastrointestinal (GI) motility by inducing GI tract contraction [4]. Mosapride works by agonistically binding to the intestinal nervous system’s 5-hydroxytryptamine type 4 (5-HT4) receptors, which increases cholinergic excitatory neurotransmission and, in turn, propulsive motility [5,6]. The term mosapride citrate comes from the fact that mosapride is a base that is more stable when it is treated with acids, most often citrate (although hydrochloric acid is also utilized). The preparation’s quality is greatly influenced by the quality of the raw materials. The level of each component in the active pharmaceutical ingredient (API) impacts the drug’s efficacy. Therefore, for salt-type medications like mosapride citrate, it is important to make sure the content of both components in the salt is correct while also selecting the right technique of quantification.

Although mosapride citrate is now excluded from the United States and Chinese pharmacopeias, it is included in the Japanese pharmacopeia [7], where the high-performance liquid chromatography (HPLC) technique is used to evaluate the drug’s concentration. Based on literature searches, three techniques have been published to date for the use of mosapride in pharmaceutical and biological samples: spectrophotometric, HPLC, and LC-MS/MS [8]. The majority of investigations on mosapride citrate raw material or synthesis have used HPLC [9,10,11,12]. However, the method using HPLC is relatively time-consuming and can only be determined by measuring the content of a fraction of mosapride citrate. It is inaccurate to use a part to calculate the overall content when the salt formation ratio is unknown.

In recent years, there have been several studies using ^1^H NMR for the quantification of analytes and the use of quantitative nuclear magnetic resonance (qNMR) has become a hot topic [13,14,15,16]. QNMR is quite suitable for identifying and measuring drugs and their related chemicals as well as identifying drugs of inferior grade. Modern qNMR techniques have developed quickly, which has resulted in the advancement of qNMR techniques, including the research of qNMR methods with external standards [17] and without the use of internal standards [18]. Currently, qNMR is still mainly used for content or purity determination [19,20]. The advantages of the qNMR method are as follows: simple, rapid, and non-destructive to the analyzed substance; the sample can be recovered after analysis; and more than one analyte in the mixture can be determined at the same time [21]. Commonly used qNMR methods include ^1^H NMR and ^19^F NMR.

Therefore, we tried to establish a simple and rapid qNMR method to analyze the content of mosapride citrate. In this study, citrate and mosapride could be quantified by internal standard separately. The differences between qNMR methods and their results were compared. Meanwhile, the salt-forming ratio of citrate and mosapride can be determined indirectly.

## 2. Results and Discussion

### 2.1. Signal Identification and Selection for Quantification

**Figure 1:**
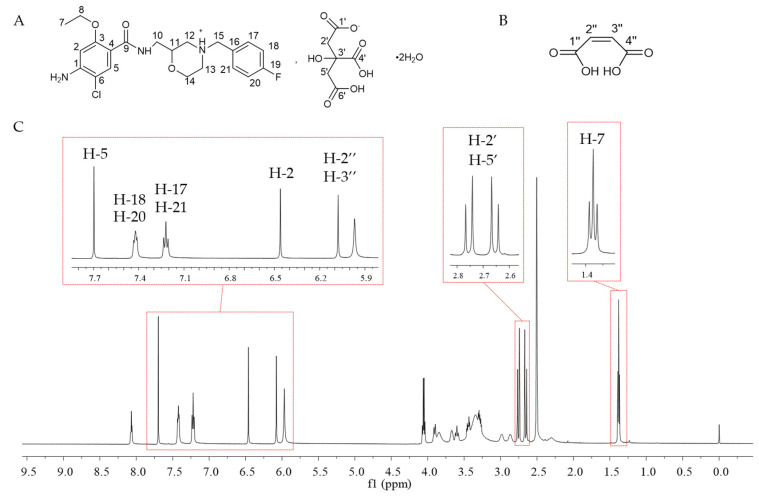
The structure of mosapride citrate * (**A**) and maleic acid (**B**), and ^1^H NMR (**C**) of mosapride citrate in DMSO-d_6_ solvent (with the internal standard). *: the structure of mosapride citrate. According to the crystal structure of mosapride citrate (deposition number: 1995115) in The Cambridge Structural Database [22], the charges of mosapride citrate were labeled.

In the quantitative analysis of ^1^H NMR, the signal peak in the analytical spectrum should be attributed first, and the number of protons corresponding to each signal peak should be clarified at the same time. Since there were multiple unwanted peaks caused by noise when automatic peak picking was used, manual peak picking was adopted for this study. Figure 1 and Figure S1 demonstrate the ^1^H NMR and ^13^C NMR spectra of mosapride citrate in DMSO-d_6_ after the addition of the internal standard. The structural assignments of peaks in ^1^H NMR and ^13^C NMR of mosapride citrate were made on the basis of 1D and 2D NMR profiles (Figures S2–S5). 

On this basis (Table 1), the appropriate signal peak should be selected as the quantitative characteristic peak. The first principle of selection was not to be interfered with by other peaks. As shown in Figure 1, the internal standard maleic acid only has a set of characteristic peaks at 6.14 ppm (2H, s). The peaks do not interfere with each other, as compared to the sample, and the baselines on both sides of the peaks were acceptable after manual baseline correction. Therefore, the peak at 6.14 ppm was selected as the internal standard quantitative peak. Mosapride has 24 protons, and the peaks without interference include H-2, H-5, H-7, H-17, H-21, H-18, and H-20. These protons were selected as candidates for the quantitative peaks of mosapride for method comparison. The citrate has only a set of proton signal peaks, so this set of proton signal peaks was chosen as the quantitative peak. The signal peak at 2.67 ppm and 2.74 ppm (4H, dd, *J* = 15.1, 58.2 Hz) of citrate was two groups of proton signals of α-CH_2_. These quantitative signals were acceptably separated from the internal standard quantitative peak and other peaks (as shown in Figure 1), which met the quantitative requirements. 

**Figure 2:**
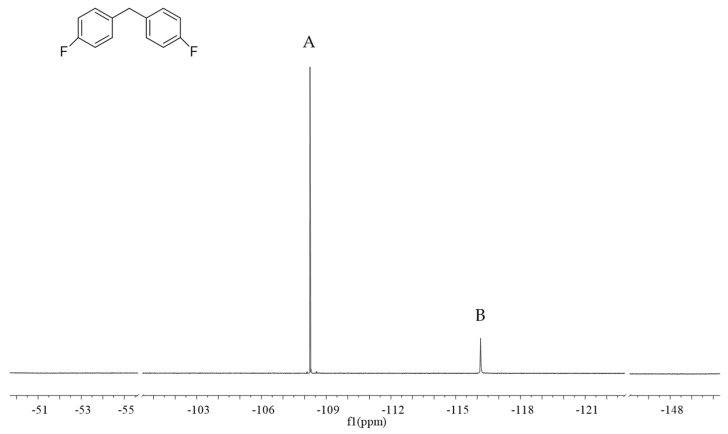
The structure of 4,4′-difluoro diphenylmethanone, and ^19^F NMR of spectra of (**A**) 4,4′-difluoro diphenylmethanone (the internal standard) and (**B**) mosapride citrate in MeOD solvent.

In the quantitative analysis of ^19^F NMR, Figure 2 demonstrates the ^19^F NMR spectra of mosapride citrate in MeOD after the addition of the internal standard, where A represents the characteristic peaks of the internal standard (4,4′-difluoro diphenylmethanone) at −108.25 ppm and B represents the characteristic peaks of mosapride citrate at −116.15 ppm. These two signals were completely separated from the internal standard quantitative peak, which met the quantitative requirements. 

### 2.2. Optimization of Deuterated Solvent and Internal Standard for qNMR

**Figure 3:**
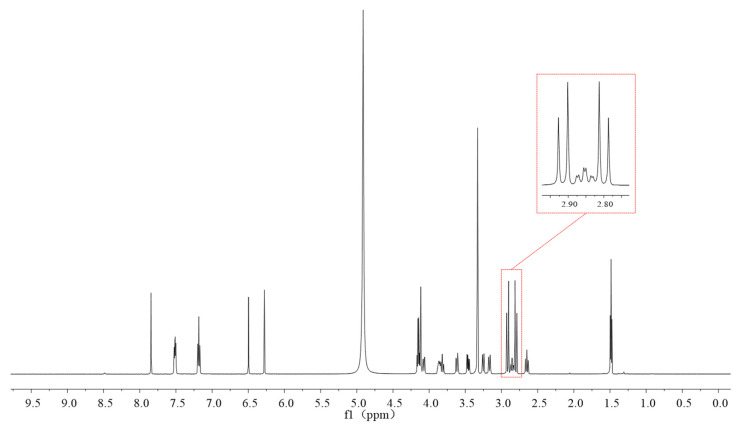
^1^H NMR of mosapride citrate in MeOD solvent.

**Figure 4:**
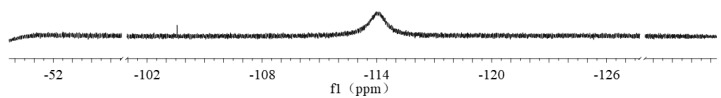
^19^F NMR of mosapride citrate in DMSO-d_6_ solvent.

The solvent affects the chemical shift in the spectrum and the position of the characteristic peak selected for quantification. We examined three solvents, which were D_2_O, DMSO-d_6_, and MeOD. When using D_2_O as the solvent, mosapride citrate was not completely dissolved, indicating that its solubility in D_2_O was poor. When using DMSO-d_6_ and MeOD as solvents, both of them could be completely dissolved. The characteristic peaks of the citrate structure were interfered with when MeOD was used as the solvent in the ^1^H NMR spectra (Figure 3). Therefore, DMSO-d_6_ was chosen as the solvent for ^1^H NMR. When DMSO-d_6_ was used as the solvent for the ^19^F NMR experimental assay, the characteristic peaks of mosapride citrate appeared to broaden (Figure 4). This may be related to solvent effects or hydrogen bonding effects, which deserve further investigation. Therefore, MeOD was chosen as the solvent for ^19^F NMR.

The internal standard should have high purity, few protons, good stability, good solubility in the deuterium solvent, have no interference with the quantitative peak of the sample, and not react with the test product and solvent. According to the chemical structure and chemical shift of mosapride citrate, maleic acid was chosen as the internal standard for ^1^H NMR and 4,4′-difluoro diphenylmethanone for ^19^F NMR. Maleic acid is acidic and can change the pH of the solution, which may lead to the occurrence of side reactions. This suggests that solution stability should be considered when selecting an internal standard that can affect the pH of the solution. Maleic acid has good solubility in DMSO-d_6_ and 4,4′-difluoro diphenylmethanone has good solubility in MeOD. The single peak of maleic acid at 6.14 ppm did not overlap with any of the signal peaks of mosapride citrate when DMSO-d_6_ was used as the solvent. The peak of 4,4′-difluoro diphenylmethanone at −108.25 ppm did not overlap with the characteristic peak of mosapride citrate when MeOD was used as the solvent. 

### 2.3. Determination of qNMR Parameters

#### 2.3.1. Temperature

Temperatures were examined at 298 K, 308 K, and 318 K. At each temperature, the selected characteristic peaks of citrate and mosapride by ^1^H NMR as well as the characteristic peaks by ^19^F NMR were integrated, and we calculated the integral area ratio with the internal standard characteristic peak area. The results are shown in Table 2 and Table S1. The final results of ^1^H NMR showed little effect of temperature on the results. Therefore, the temperature of ^1^H NMR was set to 298 K. The results of ^19^F NMR showed that the signal–noise ratio was the best at 308 K. Therefore, the temperature of 308 K was chosen for ^19^F NMR.

#### 2.3.2. D1

Relaxation delay (D1) is an important parameter in quantitative NMR. Its value is affected by the pulse angle used in the pulse program and the longest longitudinal relaxation time (T_1_) [23]. The longitudinal relaxation time T_1_ should not be too long; otherwise, it would cause shifts and deformations in the line [24]. Typically, in order to obtain the complete relaxation of the selected signal, the relaxation delay value is set to at least five times the longest T_1_ of the quantification protons (for 90 pulses) [25,26]. Many research articles have reported different recycle delay times of 10 s and 20 s to achieve the full relaxation of the magnetization intensity for recording NMR spectra [27,28]. In this study, T_1_ was calculated for the different quantitative and internal standard peaks selected and the longest T_1_ for ^1^H NMR and ^19^F NMR were 5.26 s and 0.866 s, respectively, so the theoretical D1 should be 26.3 s and 4.33 s, respectively. Therefore, 27 s was finally chosen for D1 for ^1^H NMR and 5 s for D1 for ^19^F NMR to ensure complete relaxation. 

#### 2.3.3. AQ

The acquisition time will affect the digital resolution of the spectrum, and it should match the time when the signal is completely decayed into noise. In this study, aq = 1 s, 2 s, 3 s, and 4 s were investigated, respectively. The results showed that the signal–noise ratio (S/N) all met the requirements. When aq = 2 s, the peak area ratio of ^1^H NMR tended to stabilize, so the aq for ^1^H NMR was chosen to be 2 s. When aq = 3 s, the FID decay of ^19^F NMR was complete, so the aq for ^19^F NMR was chosen to be 3 s.

#### 2.3.4. NS

Experimentally, the NS can be improved to reach an acceptable S/N equal to or greater than 250:1 for ^1^H NMR and ^19^F NMR in order to obtain accurate quantitative results. Therefore, the different number of scans (8, 16, 32, and 64) were optimized under the condition that the other parameters remain unchanged. The results showed that the S/N ratios under these scanning times were all greater than 250. When the NS was 16, the S/N was relatively large (Table 2 and Table S1). In order to ensure the accuracy of the integrated area ratio, 32 was selected as the NS of ^1^H NMR and ^19^F NMR.

### 2.4. Validation

#### 2.4.1. Precision

Precision refers to the degree of dispersion between each measurement value in multiple measurements of a certain quantity, which represents the difference between the results produced by the instrument. The precision analysis of qNMR consists of six consecutive measurements of the subject. We calculated the relative standard deviation (RSD) of the six results using the ratio of the selected characteristic peaks to the internal standard peaks. In ^1^H NMR, the RSD of citrate was 0.94% (n = 6) and the RSDs of mosapride were all less than 2% (Table S2). In ^19^F NMR, the RSD was 0.60% (n = 6). 

#### 2.4.2. Repeatability

Repeatability refers to the degree of the dispersion of measurement results under the same operating conditions. The test solution was arranged in six parallel parts, and each sample was determined once. The results showed that the RSD of citrate was 1.21% (n = 6) and the RSDs of mosapride were all less than 2% (Table S2) in ^1^H NMR. In ^19^F NMR, the RSD was 1.42% (n = 6). 

#### 2.4.3. Linearity

We varied the concentration of the sample under test to assess linearity in the range of 5–15 mg/mL. In ^1^H NMR, the mass ratio of citrate to the internal standard (or mosapride to internal standard) was taken as the abscissa (X), and the ratio of citrate to the quantitative peak area of the internal standard (or mosapride to the quantitative peak area of the internal standard) was taken as the ordinate (Y). The linear regression equation was y = 0.3715x + 0.2701 (*r* = 0.9991) for citrate and y = 0.0908x − 0.012 (*r* = 0.9994) for mosapride at 6.46 ppm. The linear correlation coefficients of the other quantitative peaks of mosapride were ≥0.999 (Table S2). The results showed that the linearity was good. 

In ^19^F NMR, the mass ratio of mosapride to the internal standard was taken as the abscissa (X), and the ratio of mosapride to the quantitative peak area of the internal standard was taken as the ordinate (Y). The linear regression equation was y = 0.2049x + 0.0044 (*r* = 0.9997). The result showed that the linearity was good.

#### 2.4.4. Stability

Mosapride citrate has good chemical stability. Due to the use of acidic internal standards and protonic solvents, stability experiments are needed. In this experiment, the stability was assessed by the continuous determination of the same samples at different times of 0, 2, 4, 6, 8, 12, and 24 h. In ^1^H NMR, the RSD of citrate was 0.50% (n = 6) and the RSDs of mosapride were all less than 2% (Table S2). In ^19^F NMR, the RSD was 1.06% (n = 6). They indicated that the sample solution was stable at room temperature for 24 h.

#### 2.4.5. Accuracy

Accuracy is generally expressed as recovery rate (%). In order to determine the accuracy of the proposed qNMR method, a known number of standard compounds are added to the sample solution for recovery experiments. Three different concentrations of low, medium, and high can be designed, and three samples of each concentration can be prepared, respectively, for evaluation. In this study, mosapride citrate control was spiked at 75%, 100%, and 125% of the target concentration into subjects with known concentration. In ^1^H NMR, the average spiked recoveries of citrate were 100.99%, and the RSDs were less than 2.21%. The recoveries of mosapride were in the range of 99.15–100.82%, and the RSDs were all less than 2.88%. They confirmed the adequate accuracy of the ^1^H NMR analytical method. In ^19^F NMR, the average spiked recoveries were 98.50%, respectively, and the RSD was 1.58%. Specific recovery results are shown in Table 3 and Table S2.

#### 2.4.6. Robustness 

In order to investigate the robustness of the method, the effects of temperature, relaxation time, and scanning times on the results were measured. Only one factor was changed, and other conditions remained unchanged. The result showed RSD < 2% (Table 4 and Table S2).

### 2.5. Comparison of Methods

Selecting different quantitative peaks will yield different quantitative results. All the characteristic peaks (H-2, H-5, H-7, H-17, H-21, H-18, and H-20) of mosapride without interference in ^1^H NMR were chosen for calculation. It should be emphasized that the quantitative peaks include single peaks and multiple peaks, and the results (Table S3) showed that there was basically no difference between the results. Therefore, choosing characteristic peaks with a smooth baseline and no interference was adequate for quantification. Mosapride citrate consists of a two-part structure, which theoretically allows for the quantitative analysis of the content of the raw material by the characteristic peaks of either the citrate or mosapride. Selecting the characteristic peaks of citrate and mosapride without interference, respectively, for comparison, the results were slightly different (Table 5). The results showed that when the peak of citrate was chosen as the quantitative peak, the calculated content of the raw material was relatively large. 

Based on the charge composition of mosapride citrate, the molar ratio of mosapride and citrate should be 1:1. The quantification of the citrate and mosapride contents by ^1^H NMR predicted that the salt-forming ratio of the two is 1:1.04 (RSD = 1.13%, n = 6). We conducted elemental analysis studies (Table S4) to determine that mosapride citrate has a salt-forming ratio of 1:0.97. The results of the above methods are generally consistent. Therefore, when using qNMR for the content determination of salt-forming compounds, it is necessary to accurately ensure the salt formation ratios of the two components.

The structure of mosapride citrate can be quantified using ^19^F NMR based on the F structure in mosapride. ^19^F NMR quantification was performed using the mosapride content to calculate the overall content, and the results (Table 5) obtained were in agreement with the results of mosapride determination in ^1^H NMR. Therefore, ^19^F NMR is also a rapid method to quantify the content of mosapride.

There are reports in the literature on the use of HPLC methods for the determination of the content of mosapride citrate. The HPLC method for the determination of mosapride citrate was based on the UV absorption of mosapride at 274 nm. Therefore, a comparison between the HPLC method and the qNMR method was carried out. The HPLC method was also used for the parallel determination of the two test samples. A total of 101.21% of the results (Table 5) were obtained by the HPLC method. The instrumental precision was also investigated, and the RSD result measured was 0.32% when one test sample was injected five times in succession. From the results, it can be seen that the difference between the qNMR method and the HPLC method for the content of mosapride citrate was not significant and the results were basically similar. 

The comparison of these methods showed that the qNMR method can be used as an alternative to HPLC for the determination of mosapride citrate. Among the different qNMR methods, the results were basically the same when the quantitative peak of mosapride was selected for determination, and the results were slightly different when the quantitative peak of citrate was selected for determination. It is hypothesized that it may be due to the difference in the salt-forming ratio between the two, and it is necessary to ensure the accurate salt-forming ratio when calculating the overall content. The ^1^H NMR method provides more comprehensive quantification, while individual qNMR methods can complement each other for better quantitative analysis.

## 3. Materials and Methods

### 3.1. Materials

A sample of mosapride citrate was purchased from Shandong New Times Pharmaceutical Co., Ltd. (Linyi, China), and stored at room temperature. Mosapride citrate (purity: 94.2%) standard was purchased from the China Academy of Food and Drug Administration (Beijing, China) and used as the reference standard (RS) for NMR signal assignment and identification. Maleic acid (purity: 99.94%, TraceCERT^®^) and 4,4′-difluoro diphenylmethanone (purity: 99.89%, TraceCERT^®^) were purchased from Sigma-Aldrich (St. Louis, MO, USA) and were used as the internal standard for quantitative analysis. Dimethyl sulfoxide-d_6_ 99.9 atom% D (DMSO-d_6_), deuterium oxide 99.9 atom% D (D_2_O), and Methanol 99.8 atom% D (MeOD) were purchased from Cambridge Isotope Laboratories (Andover, MA, USA) and used as the solvent for sample preparation. NMR tube and rubber tube cap (Norell D090721A-GE) were purchased from NORELL, Inc. (Landisville, NJ, USA). 

The reagents used in HPLC were chromatographic grade. Methanol (MeOH) was purchased from Beijing J&K Scientific Co., Ltd. (Beijing, China). The water purification machine used is an ultra-pure water machine (Fied-X) purchased from Field (Beijing) Scientific Instrument, Ltd. (Beijing, China). Potassium dihydrogen phosphate and phosphoric acid were purchased from Beijing Chemical Works Co., Ltd. (Beijing, China).

### 3.2. Sample Preparation

The solution for ^1^H NMR was prepared as follows: The working solutions of maleic acid, used as the internal standard, were prepared at 1.0 mg/mL in DMSO-d_6_ or D_2_O or MeOD. The sample solutions of mosapride citrate were prepared by approximately weighing 10 mg and diluting into 1 mL of the working solutions of the internal standard, and ultrasound-mixed to complete dissolution. The linearity curves for mosapride citrate were constructed using five concentrations within a certain range of 5–15 mg/mL (5, 7.5, 10, 12.5, and 15 mg/mL). For the spike recovery study, the sample of mosapride citrate was mixed with the reference of mosapride citrate and solvent DMSO-d_6_ in an appropriate volume to obtain mixtures spiked with mosapride citrate at 75%, 100%, or 125% test concentration levels. Each sample (600 μL) was transferred using a micropipette into a Norell D090721A-GE NMR tube (NORELL, Inc.).

The solution for ^19^F NMR was prepared as follows: The working solutions of 4,4′-difluoro diphenylmethanone, used as the internal standard, were prepared at 0.8 mg/mL in DMSO-d_6_ or MeOD. The sample solutions of mosapride citrate were prepared by approximately weighing 10 mg and diluting into 2 mL of the working solutions of the internal standard, and ultrasound-mixed to complete dissolution. The linearity curves for mosapride citrate were constructed using five concentrations within a certain range of 2.5–7.5 mg/mL (2.5, 3.75, 5, 6.25, and 7.5 mg/mL). For the spike recovery study, the sample of mosapride citrate was mixed with the reference of mosapride citrate and solvent MeOD in an appropriate volume to obtain mixtures spiked with mosapride citrate at 75%, 100%, or 125% test concentration levels. Each sample (600 μL) was transferred using a micropipette into a Norell D090721A-GE NMR tube (NORELL, Inc.).

### 3.3. NMR Parameters

All the NMR spectra were collected using a Bruker AVANCE NEO 600 MHz spectrometer at 25.0 ± 0.1 °C equipped with a Prodigy TCI cryoprobe, and autosampler of type PLC on TTY1 of ELCB 1. Spin multiplicities are reported as singlet (s), doublet (d), double doublet (dd), triplet (t), and multiplet (m). In addition, coupling constants (J) are reported in Hz. The unambiguous assignments for all the NMR signals were achieved by a combination of 1D and 2D NMR experiments, such as DEPT 135, HSQC, HMBC, and [^1^H-^1^H]-COSY, using the standard sequences of the TopSpin software (version 3.6.2, Bruker Biospin, Karlsruhe, Germany). The spectra were processed with the TopSpin version 3.6.2 and MestReNova software (version 5.3.1–4696, 2009 Mestrelab Research, S.L, Santiago, Spain). 

The ^1^H NMR spectra were acquired in 32 scans using 48,074 data points and zerofilled to 64k data points by the zg30 pulse program. Two dummy scans were performed prior to the experimental data collection. The NMR probe was maintained at 298 K during the whole experiment. The pulse sequence for measuring T_1_ was t1irpg. The highest T_1_ value obtained for the signal of interest was 5.26 s. The acquisition time (AQ) was equal to 2.0 s and the D1 was set to 27 s. The spectra were processed with 65,536 points using an exponential multiplication of 0.3 Hz, a manual phase correction, a manual baseline correction, and a manual integration.

All the ^19^F NMR spectra were recorded in the range of δF −50.0~−150.0 ppm (4,4′-difluoro diphenylmethanone as reference, δ = −108.2 ppm) with center frequency (O1P) at −100.0 ppm, by the zgfhiggn.2. pulse program. The NMR probe was maintained at 308 K during the whole experiment. The pulse sequence for measuring T_1_ was t1irpg. The highest T_1_ value obtained for the signal of interest was 0.866 s, so D1 of 5 s was adopted to ensure full T_1_ relaxation. The acquisition time (AQ) was set to 3.0 s. Manual shimming was acquired to obtain a homogeneous magnetic field and data were processed using the TopSpin software with manual baseline, phase correction, and integration.

For data processing, several studies [21,29,30] have shown that the automated procedure of the MestReNova software produced slightly incorrect phase and baseline corrections, and accurate results can be obtained by extremely careful manual spectral correction and manual integration. Therefore, in this study, manual phase correction integration was used for the integration process and was completed in one day. The choice of integrating the quantitative peaks from the tangent of the peaks and the uniform data processing method resulted in more accurate results.

### 3.4. Quantitative NMR Calculation Method

The internal standard method is the addition of an internal standard to quantify the quantity of the test drug, and the quantification is usually obtained from the ratio of the specific signal of the analyte to the integral value of the internal standard [31]. The amount of mosapride citrate was calculated from the following equation [32]:

$$W_{S}=W_{r}\times \frac{A_{s}}{A_{r}}\times \frac{E_{s}}{E_{r}}$$

W_s_ and W_r_ are the mass of the sample and the internal standard substance, respectively; A_s_ and A_r_ are the response signal areas of the sample and internal standard, respectively; and E_s_ and E_r_ are the molecular equivalent weights of the sample and internal standard, respectively (it is calculated by dividing molecular weight by the numbers of protons at corresponding characteristic peaks). 

The molar ratio of the two components was used for calculating the salt-forming ratio of mosapride citrate, which was determined as follows:

$$salt\text{-}forming\;ratio=\frac{A_{mosapride}}{N_{mosapride}}:\frac{A_{citrate}}{N_{citrate}}$$

A_mosapride_ and A_citrate_ are the response signal areas of mosapride and citrate, respectively; and N_mosapride_ and N_citrate_ are the numbers of protons at corresponding characteristic peaks of mosapride and citrate, respectively.

### 3.5. HPLC Analysis

A sample of mosapride citrate 0.01 mg/mL was prepared, fixed in 70% methanol, and prepared in two parallels for determination. The same concentration of mosapride citrate standard was used and prepared in the same way. Determination was carried out using the external standard method. 

The HPLC system is composed of LPG-3400SD pumps, a VWD-3x00RS Variable Wavelength Detector, and a WPS-3000RS Autosampler and TCC-3x00RS Column Compartment (all from Thermo Fisher Inc., Seattle, WA, USA). A reversed-phase Symmetry C18 (SHISEIDO Co., Ltd., Tokyo, Japan) column (250 mm × 4.6 mm i.d.; particle size 3 μm) was used for separation. The chromatographic and the integrated data were recorded using Chromeleon 7.2 SR5 (Thermo Fisher, USA) computer system. 

The mobile phase was 0.02 M monopotassium phosphate–methyl alcohol (30:70, *v*/*v*), and the buffer pH was adjusted to 4.0 with phosphoric acid. Before delivering into the system, it was filtered through a 0.45 μm PTFE filter and degassed using a vacuum. The analysis was carried out under isocratic conditions using a flow rate of 1.0 mL/min at room temperature (40 °C). Chromatograms were recorded at 274 nm using a VWD-3x00RS Variable Wavelength detector. 

### 3.6. Elemental Analysis

An elemental analyzer (UNICUBE, Elementar, Germany) was used for CHN and O elemental analysis. The samples, in powder form, were dried overnight in an oven at 60 °C before the measurement to ensure the complete removal of any residual water. A total of 1.5–2.5 mg samples were sealed in tin capsules. The samples were immediately transferred to the automatic sampler of the elemental analyzer. The combustion was carried out at 1800 °C using pure oxygen as carrier gasses.

## 4. Conclusions

In this experiment, the effects of different qNMR quantitative methods on the determination of the content of mosapride citrate were compared; a fast, accurate, and simple method for the determination of mosapride citrate using ^1^H NMR and ^19^F NMR was established; and its precision and accuracy were verified. The advantage of this method is that it is simple to operate, does not require the use of standard reagents and other reagents in practical applications, and does not involve a complicated pretreatment process. This study provides a basis for the selection of qNMR methods. Meanwhile, the salt-forming ratios of citrate and mosapride can be predicted by quantifying their contents by ^1^H NMR, respectively. It is expected that in the compound preparation of mosapride citrate, the contents of citrate and mosapride can be determined directly, or the total content can be calculated by determining a part of them according to their salt-forming ratios. Between each qNMR method, the accuracy of the method can be guaranteed, so the appropriate qNMR method can be selected for the quantitative analysis of compounds according to their properties.

## Figures and Tables

**Table 1:** ^1^H NMR and ^13^C NMR signal attribution of mosapride citrate.

NO.	*δ*_H_, multiplicity (*J* in Hz)	*δc*
1	-	149.17
1-NH_2_	5.99, s	-
2	6.46, s	98.70
3	-	157.20
4	-	110.35
5	7.70, s	132.06
6	-	109.54
7	1.38, t (6.9)	14.88
8	4.06, q (6.9)	64.92
9	-	164.31
9-HN	8.08, t	-
10	3.32, 3.48, m	43.15
11	3.74, s	72.91
12	3.18, s	53.87
13	3.06, s	51.22
14	3.64, q (8.3, 4.6)	64.92
15	4.12, s	59.88
16	-	162.90
17, 21	7.28, t (8.6)	116.12, 115.98
18, 20	7.48, dd (5.9, 8.5)	133.64
19	-	160.30
1′, 6′	-	171.75
2′, 5′	2.67, 2.74, dd (15.1, 58.2)	43.15
3′	-	72.91
4′	-	175.01
1″, 4″	-	167.40
2″, 3″	6.14, s	133.64

**Table 2:** S/N and peak area ratio of mosapride and citrate to internal standard maleic acid under different NMR parameters.

Parameters	Set Value	^1^H NMR				^19^F NMR	
		A_s1_*/A_r1_*	S/N^1^	A_s2_*/A_r1_*	S/N^2^	S/N	A_s_/A_r2_*
T	298 K	4.0065	2667.78	0.9285	2210.58	464.53	1.0442
	308 K	4.0281	2877.62	0.9200	2339.72	556.57	1.0521
	318 K	4.0163	3252.35	0.9167	2539.84	483.82	1.0218
NS	8	4.0764	1671.05	0.9634	1404.71	272.33	1.0678
	16	3.9753	2263.23	0.9314	1902.72	379.22	1.0477
	32	3.9120	3142.23	0.9324	2636.65	558.89	1.0492
	64	3.9604	4203.56	0.9357	3509.34	753.59	1.0631

S/N^1^: quantitative peak SNR of citrate at in ^1^H NMR; S/N^2^: quantitative peak SNR of mosapride in ^1^H NMR; A_s1_*: quantitative peak area of citrate in ^1^H NMR; A_r1_*: quantitative peak area of maleic acid in ^1^H NMR; A_s2_*: quantitative peak area of mosapride at 6.46 ppm in ^1^H NMR; A_r2_*: quantitative peak area of 4,4′-difluoro diphenylmethanone in ^19^F NMR.

**Table 3:** Recovery results of the quantitative nuclear magnetic resonance (qNMR) method for the mosapride citrate raw material (mean ± SD, n = 3).

Method	Concentration Level	Recovery (%)
^1^H NMR (citrate)	Low (7.5 mg/mL)	101.96 ± 0.40
	Middle (10 mg/mL)	102.31 ± 1.19
	High (12.5 mg/mL)	98.72 ± 2.21
^1^H NMR (mosapride *)	Low (7.5 mg/mL)	101.66 ± 1.37
	Middle (10 mg/mL)	100.32 ± 0.69
	High (12.5 mg/mL)	100.18 ± 0.24
^19^F NMR	Low (7.5 mg/mL)	97.72 ± 1.23
	Middle (10 mg/mL)	100.22 ± 0.53
	High (12.5 mg/mL)	97.57 ± 1.09

*: the results of mosapride at 6.46 ppm.

**Table 4:** Robustness assessment for the qNMR method for the mosapride citrate raw material.

Parameter Target Value	Modified Parameters	^1^H NMR		^19^F NMR
		RSD (citrate)	RSD (Mosapride *)	RSD
T = 298 K	T = 296 K	1.73%	0.47%	1.78%
	T = 300 K			
D1 = 27 s	D1 = 25 s	0.50%	0.73%	1.57%
	D1 = 29 s			
NS = 32	NS = 30	0.42%	0.56%	0.59%
	NS = 34			

*: the results of mosapride at 6.46 ppm.

**Table 5:** Results of different qNMR methods and the HPLC method for the mosapride citrate raw material (mean ± SD, n = 6).

Method	^1^H NMR (Citrate *)	^1^H NMR (Mosapride *)	^19^F NMR	HPLC
Content (%)	104.22 ± 1.21	99.24 ± 0.98	99.82 ± 1.42	101.62 ± 1.07

Citrate *: calculation of the overall content with the characteristic peak of citrate. Mosapride *: calculating the overall content with the characteristic peak of mosapride at 6.46 ppm.

## Data Availability

The datasets and materials used and/or analyzed during the current study are available from the corresponding author upon reasonable request.

## Supplementary Materials

The following supporting information can be downloaded at: https://www.mdpi.com/article/10.3390/ijms251910442/s1.
