# Supplementary material for: Comparison and Determination of the Content of Mosapride Citrate by Different qNMR Methods
Source: Int J Mol Sci. 2024 Sep 27;25(19):10442. doi: 10.3390/ijms251910442 (PMC11476420; doi:10.3390/ijms251910442)
Supplement: Supplementary file 1 [file ijms-25-10442-s001.zip › ijms-3119437-supplementary.pdf]

International Journal of *Molecular Sciences*

MDPI

Supplementary Material

Comparison and determination of the content of mosapride citrate by different qNMR methods

Xiaofang Lian[1,†], Yiran Li[1,†], Limin Zuo[1], Xuejia Zhao[1], Huiyi Liu[1], Yongsheng Gu[1,2], Qingying Jia[1], Jing Yao[3*] and Guangzhi Shan[1*]

[1] Institute of Medicinal Biotechnology, Chinese Academy of Medical Sciences & Peking Union Medical College, Beijing 100050, China; xiaofanglian0365@163.com (X.L.); liyiran@imb.pumc.edu.cn (Y.L.); zuo0607@163.com (L.Z.); zhaoxuejiajia@163.com (X.Z.); huiyiliu1227@163.com (H.L.); gys0525 @163.com (Y.G.); qyjia1130@163.com (Q.J.)

[2] College of Pharmacy, Xinjiang Medical University, Urumqi 830017, China

[3] Institute for the Control of Chemical Drugs, National Institutes for Food and Drug Control, Beijing 100001, China

* Correspondence: yaojh@nifc.org.cn (J.Y.); shanguangzhi@imb.pumc.edu.cn (G.S.); Tel.: +86-010-53851516 (J.Y.); Tel.: +86-010-67019851 (G.S.)

† These authors contributed equally to this work.

Table S1. S/N and peak area ratio of different mosapride quantitative peaks to internal standard maleic acid under different NMR parameters

Parameters	Set value	H-2 (δ_H 6.46)		H-5 (δ_H 7.70)		H-7 (δ_H 1.38)		H-17, H-21 (δ_H 7.28)		H-18, H-20 (δ_H 7.48)		All signals without interference
		A_{s1}^*/A_{r1}^*	S/N	A_{s2}^*/A_{r1}^*	S/N	A_{s3}^*/A_{r1}^*	S/N	A_{s4}^*/A_{r1}^*	S/N	A_{s5}^*/A_{r1}^*	S/N	A_{s6}^*/A_{r1}^*
T	298K	0.9285	2210.58	0.9161	2996.34	2.7307	3330.57	1.8572	1214.84	1.8585	892.43	8.2910
	308K	0.9200	2339.72	0.9187	3018.71	2.7604	3538.29	1.8904	1419.37	1.8855	976.04	8.3749
	318K	0.9167	2539.84	0.9296	3365.30	2.7613	3952.31	1.8748	1540.71	1.8622	997.90	8.3444
NS	8	0.9634	1404.71	0.9771	1869.87	2.8383	2058.83	1.9630	700.60	1.9556	493.21	8.6972
	16	0.9314	1902.72	0.9464	2532.57	2.7722	2800.83	1.9101	952.45	1.8904	669.50	8.4504
	32	0.9324	2636.65	0.9332	3437.32	2.7202	3918.48	1.8650	1351.93	1.8538	954.37	8.3045
	64	0.9357	3509.34	0.9317	4438.36	2.7776	5267.57	1.8812	1900.65	1.8592	1366.18	8.3853

A_{r1}^*: quantitative peak area of maleic acid in ^{1}H NMR; A_{s1}^*: quantitative peak area of mosapride at 6.46 ppm in ^{1}H NMR; A_{s2}^*: quantitative peak area of mosapride at 7.70 ppm in ^{1}H NMR; A_{s3}^*: quantitative peak area of mosapride at 1.38 ppm in ^{1}H NMR; A_{s4}^*: quantitative peak area of mosapride at 7.28 ppm in ^{1}H NMR; A_{s5}^*: quantitative peak area of mosapride at 7.48 ppm in ^{1}H NMR; A_{s6}^*: the total of quantitative peak area of mosapride in ^{1}H NMR.

Table S2. Verification results of different mosapride quantitative peaks for determination of mosapride content

Signal (δ_H, ppm)	Precision (RSD, n=6)	Repeatability (mean ± SD, n=6)	Linearity	Stability (RSD)	Accuracy (mean ± SD, n=9)	Robustness (RSD, n=3)		
						T	D1	NS
7.70	0.41%	64.70%±0.76%	y=0.0843x+0.0273 (r=0.9993)	0.64%	99.15%±2.05%	0.79%	1.53%	0.68%
7.48	1.04%	64.42%±0.62%	y=0.1789x-0.0462 (r=0.9993)	0.71%	99.38%±2.12%	1.02%	1.00%	1.19%
7.28	1.19%	64.90%±	y=0.1822x-0.047	0.80%	100.82%±1.86%	1.54%	1.45%	1.41%

		1.16%	9 (*r*=0.9995)					
6.46	0.71%	64.28%±0.70%	y=0.0908x-0.012 (*r*=0.9994)	0.86%	100.72%±1.05%	0.47%	0.73%	0.56%
1.38	0.61%	65.29%±1.20%	y=0.2571x+0.1347 (*r*=0.9991)	1.18%	99.42%±2.88%	0.47%	0.77%	0.81%
All signals without interference	0.57%	65.12%±0.50%	y=0.7933x+0.0558 (*r*=0.9996)	0.80%	99.84%±0.94%	0.55%	0.58%	0.51%

Table S3. Results of selecting different quantitative peaks in qNMR methods for the mosapride (mean ± SD, n=6)

Quantitative peak of mosapride	H-2 (δH 6.46)	H-5 (δH 7.70)	H-7 (δH 1.38)	H-17, H-21 (δH 7.28)	H-18, H-20 (δH 7.48)	All signals without interference
Content (%)	99.24 ± 0.98	100.01 ± 1.13	101.89 ± 1.74	100.02 ± 1.81	99.39 ± 1.16	100.78 ± 1.22

Table S4. Results of elemental analysis

Element	C	H	O	N
content (%)	51.52	5.26	34.26	6.75
atomic number	26.83	32.89	13.38	3.01

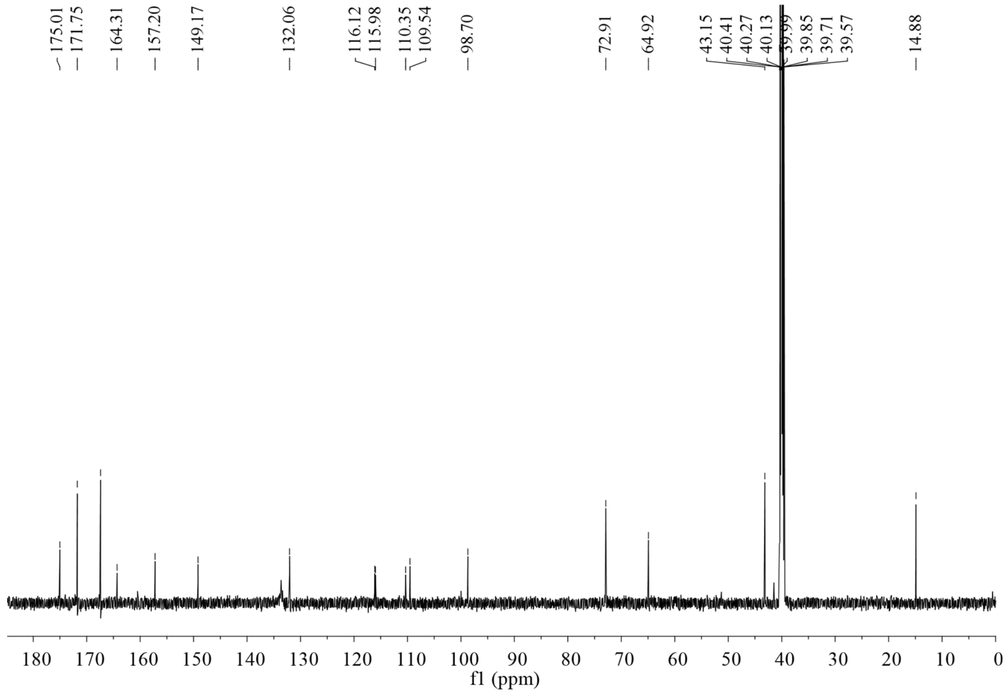

Figure S1. ^{13}C NMR spectrum of mosapride citrate in DMSO-d_6 solvent (with the internal standard)

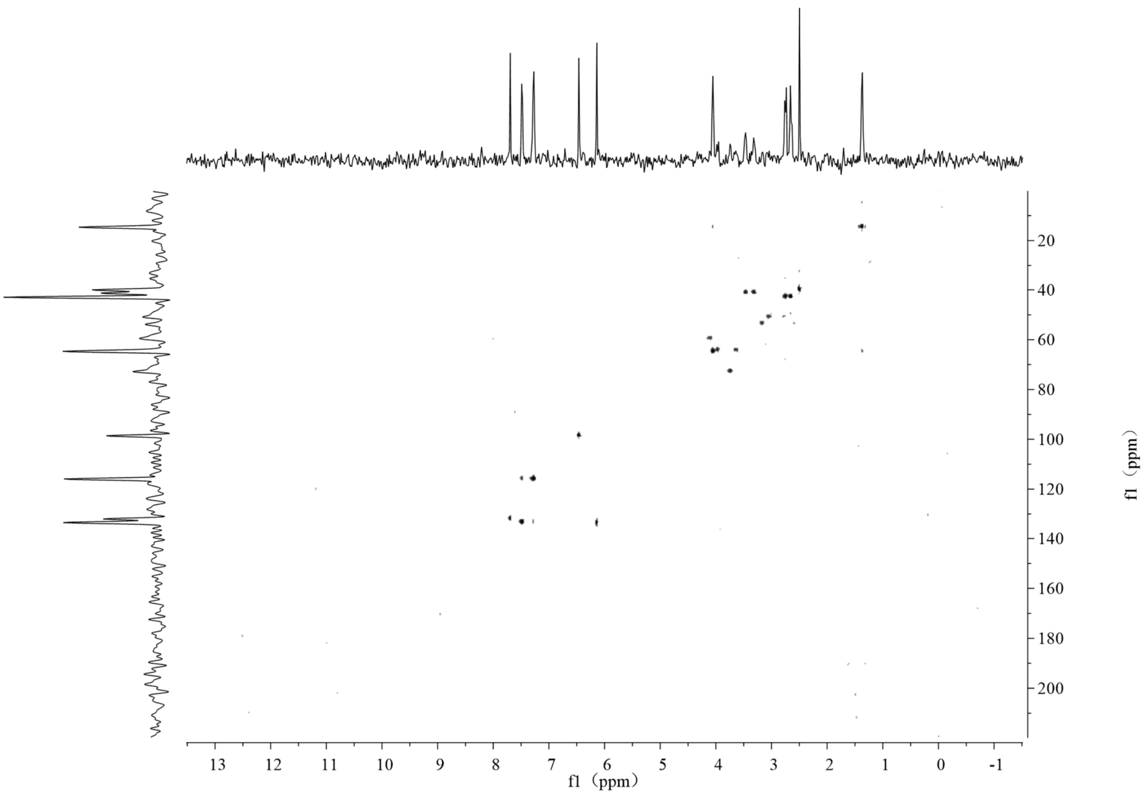

Figure S2. HSQC spectrum of mosapride citrate in DMSO-d_6 solvent (with the internal standard)

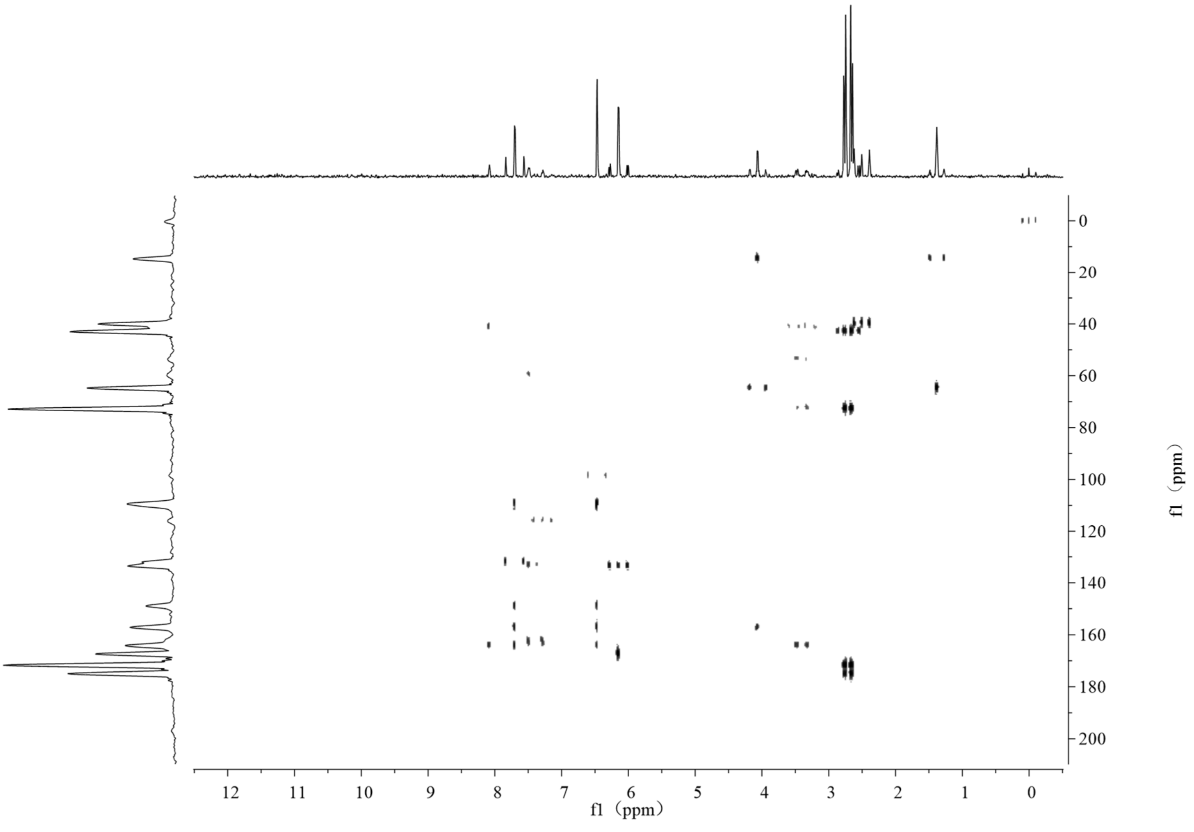

Figure S3. HMBC spectrum of mosapride citrate in DMSO-d_6 solvent (with the internal standard)

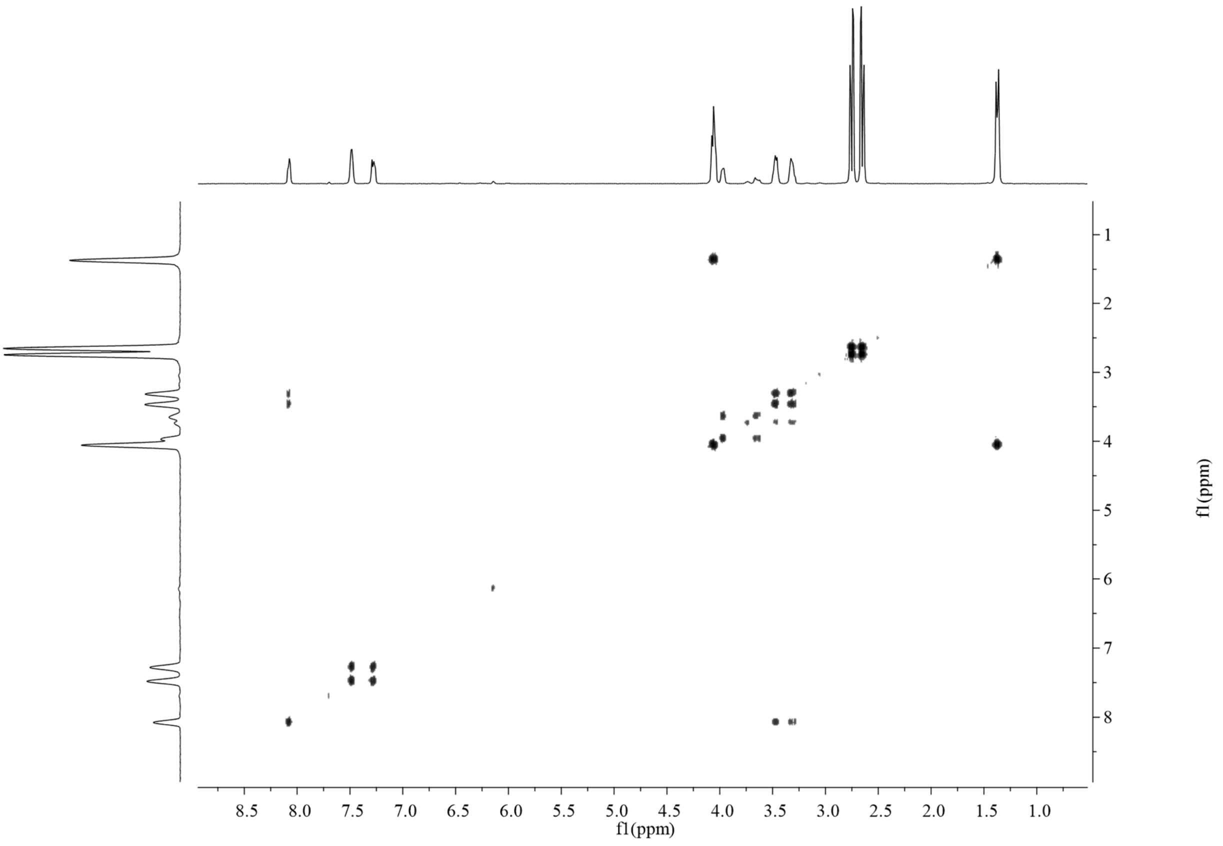

Figure S4. [^{1}H-^{1}H]-COSY spectrum of mosapride citrate in DMSO-d6 solvent (with the internal standard)

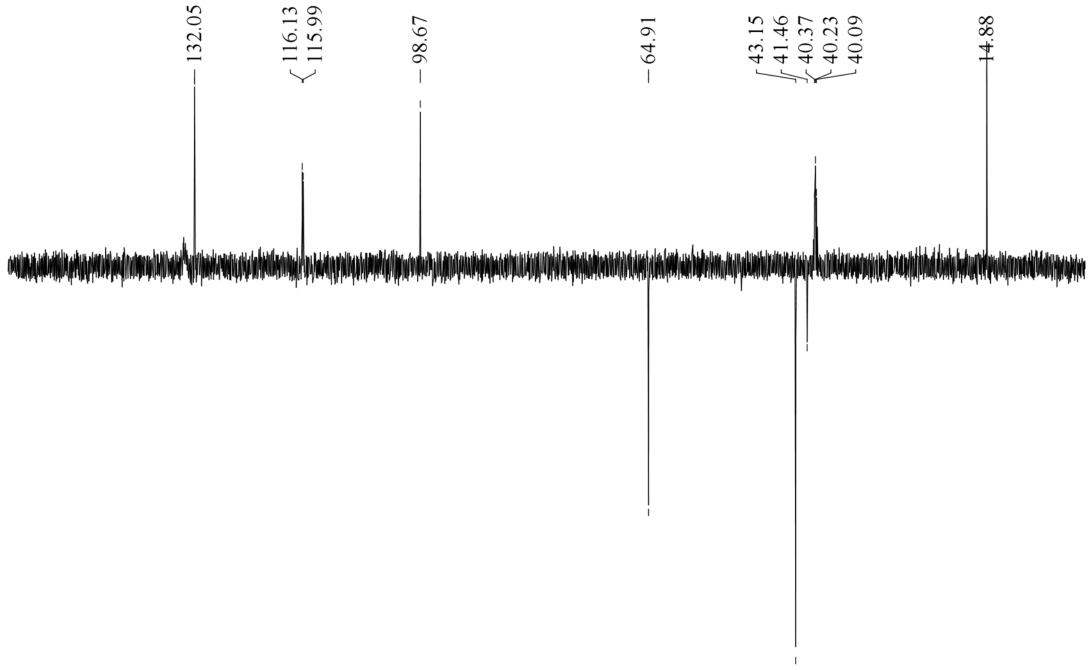

Figure S5. DEPT spectrum of mosapride citrate in DMSO-d6 solvent (with the internal standard)
